# Thermal-Hydraulic Performance Optimization of Open Microchannel Heat Sinks with Integrated Front Triangular Ribs and Secondary Channels

**DOI:** 10.3390/mi17091052

**Published:** 2026-09-03

**Authors:** Jianlong Gong, Zhongliang Pan

**Affiliations:** 1 School of Electronics Science and Engineering, South China Normal University, Foshan 528225, China; gongjianlong@gdcp.edu.cn; 2 School of Mechanical and Electrical Engineering, Guangdong Communication Polytechnic, Guangzhou 510650, China

**Keywords:** open microchannel heat sinks, open front triangular ribs, open secondary channels, thermal-hydraulic performance, response surface methodology, genetic algorithm

## Abstract

To enhance the comprehensive thermal-hydraulic performance of microchannel heat sinks (MCHS), this work proposed a novel open-type MCHS integrated with front triangular ribs and secondary channels. Firstly, numerical computations were carried out to compare the flow and heat transfer characteristics of seven distinct MCHS configurations, and identify the optimal hybrid geometry consisting of an open microchannel fitted with integrated front triangular ribs and secondary channels. Secondly, the effects of *H*_1_ (height of open front triangular ribs) and *H*_2_ (height of open secondary channels) on the thermal-hydraulic performance of the MCHS were systematically analyzed. The results revealed that the composite open MCHS obtained its peak performance evaluation criterion (*PEC*) value at *H*_1_ = 0.15 mm and *H*_2_ = 0.25 mm from separate single-factor parametric analyses for *H*_1_ and *H*_2_*,* at a mass flow rate of 0.9 g/s. Finally, a surrogate model correlating three structural parameters (*H*_1_, *H*_2_, *W*_w_) with the optimization objective (*PEC*) was established based on orthogonal test data. This surrogate model was coupled with a genetic algorithm to perform global parameter optimization to maximize *PEC*. Relative to the original design and the reference geometry in other literature, the optimized design achieved maximum reductions of 15.3% in thermal resistance and 70.9% in pumping power, accompanied by a 55.7% improvement in *PEC*. This research provides theoretical support for the structural design of high-heat-flux microchannel cooling devices.

## 1. Introduction

Driven by the continuous iteration of microelectronic manufacturing techniques, integrated chips with ultra-high computing density have been extensively deployed across electronic cooling scenarios. The continuous upgrade of chip functional modules leads to a sharp surge in internal heat generation, which makes efficient thermal control an indispensable core constraint restricting the stable and long-term operation of microelectronic devices. Benefiting from a compact structural configuration and outstanding heat exchange capacity, microchannel heat sinks (MCHS) are recognized as a feasible cooling solution to address ultra-high heat flux loads of miniaturized chips. However, conventional straight rectangular microchannels adopt a uniform channel cross-section design with simple processing characteristics, while obvious drawbacks including uneven surface temperature distribution and excessive temperature gradient restrict their comprehensive cooling capacity [1]. Reasonable adjustments to microchannel cross-sectional geometry, fluid flow path, channel configuration mode and inlet placement can optimize multiple critical thermal-hydraulic indicators, including average substrate temperature, pressure drop, heat transfer coefficient, Nusselt number and surface temperature uniformity [2]. To maximize the heat transfer capability of MCHS, numerous scholars have attempted performance enhancement through geometric modification strategies. Among these strategies, altering the cross-sectional profiles of microchannels is a widely adopted approach [3,4,5,6,7,8]. Other common measures include embedding micro-fins, micro-pits and micro-grooves, etc. [9,10,11,12,13,14,15,16,17,18]. However, such geometric improvements have effectively reduced thermal resistance while inevitably exacerbating pressure drop.

To achieve improved cooling capacity accompanied by restrained fluid pressure drop, some researchers have studied microchannel structures with fins and secondary flow channels. Wen’s research group adopted numerical simulation methods to compare the thermal-hydraulic characteristics of five composite structures integrating secondary channels and different shaped ribs, covering rhombus, rectangle, rear triangle, front triangle and ellipse configurations [19]. Their comparative analysis verified that the matching scheme consisting of front triangular ribs and auxiliary secondary channels delivers the optimal heat transfer capacity among all tested structures. Further parametric exploration targeting rib geometric dimensions and secondary channel width was also carried out in their work. Under the flow condition of *Re* = 200, the optimized composite structure achieved a 34% improvement in comprehensive thermal performance relative to ordinary straight MCHS. To improve the limited thermal-hydraulic performance of microchannel structures with a single cooling feature, Dawood et al. [20] conducted comparative numerical simulations on four typical configurations: conventional straight rectangular microchannels, single front triangular rib microchannels, independent secondary-channel microchannels and the composite configuration integrating front triangular ribs and auxiliary secondary channels. Their comparative data confirmed that the combined configuration of front triangular ribs and secondary channels achieved optimal thermal-hydraulic performance among all tested schemes. Furthermore, additional parametric research on the rib arrangement spacing λ was carried out in their work. Under the flow condition of Re = 1000 with λ fixed at 2, this composite structural design reached its peak overall cooling performance. Given the contradictory relationship between heat transfer augmentation and rising flow loss in MCHSs, Chen et al. [21] developed an original microchannel configuration fitted with built-in ribs and secondary flow passages. Comprehensive numerical analyses were performed to reveal the individual effects of rib geometric size, quantity and outline shape on thermal-hydraulic behaviors. By integrating second-order response surface models with the NSGA-II multi-objective genetic algorithm, they carried out parameter optimization of normalized rib length and width. Their computational data indicated that the optimized composite configuration lowered pumping power by 31.6% and improved the overall thermal-hydraulic performance criterion by 16.7%. To tackle the limited heat transfer intensification capacity of traditional ribbed MCHS, Fan et al. [22] proposed a unique cooling structure equipped with cylindrical oblique ribs and auxiliary secondary channels for numerical investigation, revealing prominent performance improvement trends of the composite configuration. Numerical contrast results demonstrated that the novel composite structure produced a 75.6% average increase in the Nusselt number and a 59.1% reduction in thermal resistance when compared with conventional straight microchannels fitted with single ribs. To explore how auxiliary flow structures regulate the thermal-hydraulic characteristics of wavy microchannels under diverse flow states, Karthikeyan and colleagues [23] conducted quantitative analyses to analyze the influences of secondary flow on flow resistance and convective heat transfer. Their computational results proved that the configuration with auxiliary channels produced remarkable heat transfer enhancement while effectively reducing pressure drop. When flow perturbing structures are arranged inside channels or local flow trajectories are redirected, secondary flow can induce swirling vortex structures. These vortices amplify turbulent fluctuations of the coolant and restrain the steady development of thermal boundary layers. Such physical mechanisms reduce the wall-adjacent temperature gradients and ultimately achieve heat transfer enhancement [24,25]. Kuppusamy’s research team [26] analyzed the thermal-hydraulic characteristics of MCHS fitted with crosswise inclined auxiliary channels arranged between adjacent flow channels. Numerical comparison showed that such secondary-channel structures achieved a 146% improvement in overall cooling performance against conventional plain microchannels, along with a 76.8% drop in thermal resistance and a slight 6% reduction in pressure loss. A. Y. Wang et al. [27] designed a novel microchannel heat sink (MCHS) equipped with triangular concave cavities and M-shaped pin fin arrays. They adopted a composite optimization framework integrating Box–Behnken design (BBD) and genetic algorithms to conduct comprehensive parametric optimization of the microchannel geometric dimensions. Their numerical outcomes demonstrated that the optimized configuration achieved a heat transfer augmentation coefficient up to 1.84. Q. F. Zhu et al. [28] carried out comprehensive investigations into the influences of fin contour, dimension and configuration distribution on the coupled thermal and hydraulic behaviors inside MCHS. Their research results indicated that fin shapes as well as their spatial placement exerted decisive impacts on balancing heat transfer enhancement and reducing pressure drop. Q. F. Zhu et al. [29] explored the thermal-hydraulic behavior of MCHS incorporating teardrop-shaped recesses and pin-fin arrays. Their analysis revealed that the ribbed column structures promoted convective heat transfer through intensified flow turbulence, whereas the recessed cavities reduced pressure drop by expanding the effective cross-sectional flow area. Synergistically integrating teardrop-profiled ribs with cavity features led to a marked improvement in comprehensive heat sink performance. The cooling capacity of MCHSs equipped with auxiliary flow channels composed of integrated pits and disturbance fins was investigated by Japar et al. [30]. According to their analysis, these secondary flow structures improved the comprehensive thermal performance by disrupting boundary-layer continuity and accelerating coolant mixing inside the primary flow domain. Paramanandam et al. [31] analyzed the thermal-hydraulic behaviors of MCHS incorporating fins, recessed cavities and auxiliary flow channels. Their research indicated that the synergistic effect of three structural elements, namely fins, cavities and secondary channels, significantly improved heat transfer capacity while increasing flow resistance. It should be noted that the above research mainly focuses on MCHS with closed fins and closed secondary channels, without considering variations in rib height and secondary channel height, and the top surfaces of ribs and secondary channels are usually in contact with the cover plate of the heat sink. Limited studies have been reported concerning open MCHS. If the heights of the fins and the secondary flow channels in the microchannel are relatively small, an open MCHS will be formed, thereby creating open spaces between the top surfaces of the ribs, the secondary flow channels and the heat sink cover plate. Prajapati et al. [32] conducted a parametric study on rectangular parallel microchannels featuring adjustable fin heights. Their results revealed that open-type configurations with fin heights ranging from 75% to 80% of the channel depth delivered superior thermal performance relative to fully enclosed counterparts. Prabhakar Bhandari et al. [33] examined the thermal-hydraulic behaviors of open MCHS equipped with square cross-section pin fins. A comparative evaluation was performed across seven distinct fin-height configurations. Their experimental findings identified 1.5 mm as the fin height yielding the highest thermal performance under the tested conditions. Yin et al. [34] demonstrated that open MCHS not only improved convective heat transfer substantially but also markedly stabilized flow boiling performance by reducing hydrodynamic instabilities. Jiang et al. [35] improved the standard closed rectangular microchannel and proposed an open-configuration design, and then performed a comprehensive analysis on the thermal-hydraulic effects induced by different turbulence-inducing fin shapes. They adopted a multi-objective genetic algorithm to seek the optimal geometric parameters. Simulation comparisons showed that the optimized configuration reduced the peak temperature of the substrate by 1.561%, and the pressure drop decreased by 51.138% compared with the original unoptimized model. Open MCHS have demonstrated their advantages for single-phase coolant flow and flow boiling. However, research on open MCHS integrated with composite secondary channels and fins remains extremely limited. A comprehensive review of the above-mentioned literature reveals that existing relevant studies predominantly focus on closed MCHS. Existing studies have not investigated the synergistic heat transfer enhancement effect of the composite structure consisting of front open triangular ribs and open secondary channels in MCHS. The synergistic heat dissipation performance of different structural combinations and the influence characteristics of structural parameters remain unclear. Compared with the existing research, the open composite structure proposed in this paper provides a novel strategy to improve the overall thermal-hydraulic performance of MCHS.

In this paper, numerical simulations were carried out on seven configurations of MCHS to compare and analyze their thermal-hydraulic performance, and the optimal composite structure is obtained. This paper has analyzed the effects of the height of front open triangular ribs and the height of open secondary channels on thermal-hydraulic performance. Combined with response surface methodology and a genetic algorithm, an optimization prediction model was established to obtain the combination of structural parameters that maximized PEC, providing a theoretical reference for the structural optimization of MCHS.

## 2. Model Description of Microchannel Heat Sinks

### 2.1. Physical Model

**Figure 1:**
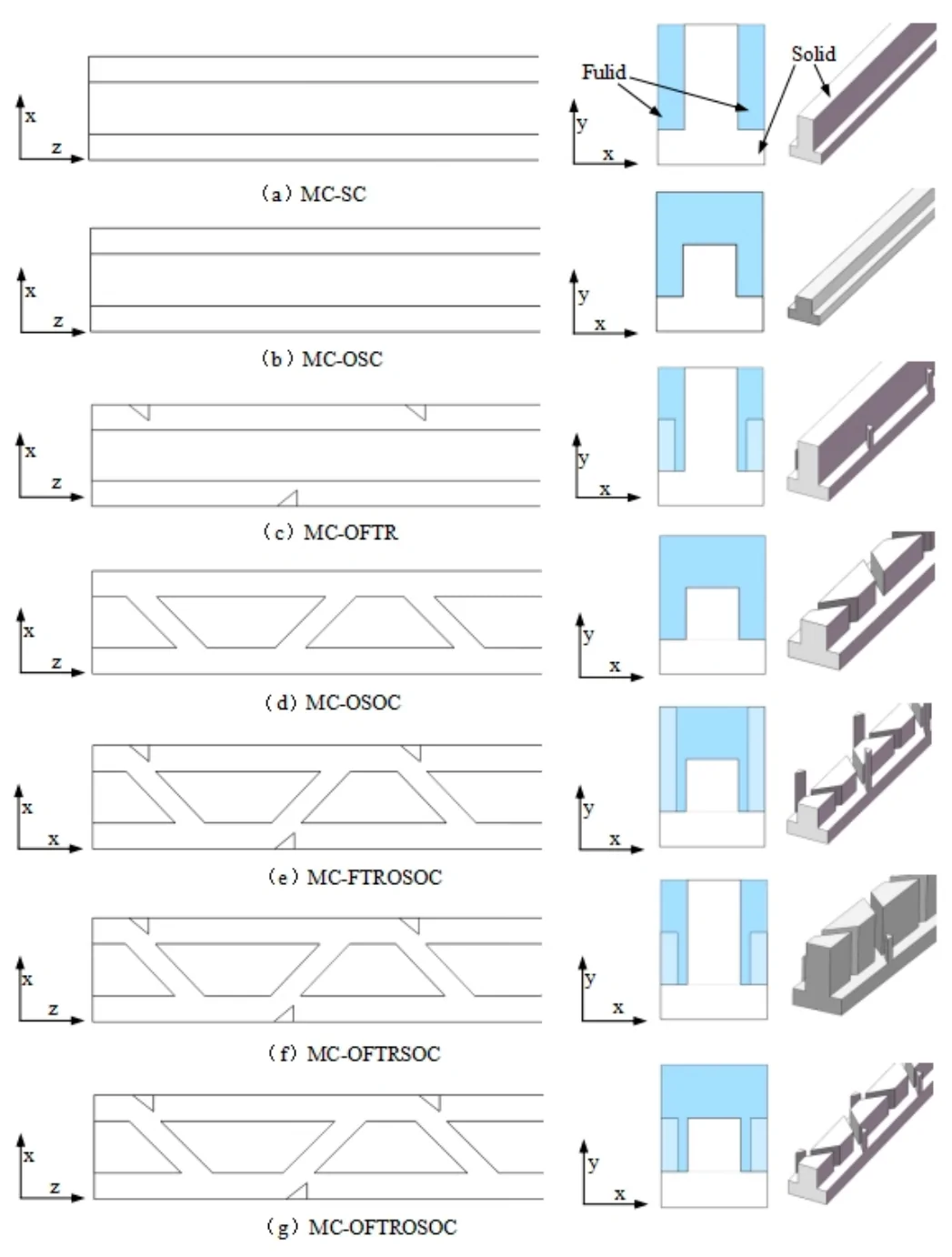
Schematic diagram of microchannels with different geometric configurations.

This study adopted seven different MCHS models for thermal-hydraulic performance evaluation, namely straight rectangular closed microchannels (MC-SC), straight rectangular open microchannels (MC-OSC), microchannels with open front triangular ribs (MC-OFTR) and microchannels with open secondary channels (MC-OSOC). The other three composite configurations were microchannels with closed front triangular ribs and open secondary channels (MC-FTROSOC), microchannels with open front triangular ribs and closed secondary channels (MC-OFTRSOC) and microchannels with open front triangular ribs and open secondary channels (MC-OFTROSOC) [19]. Figure 1 presents the geometric schematics of the seven proposed MCHS structures.

**Figure 2:**
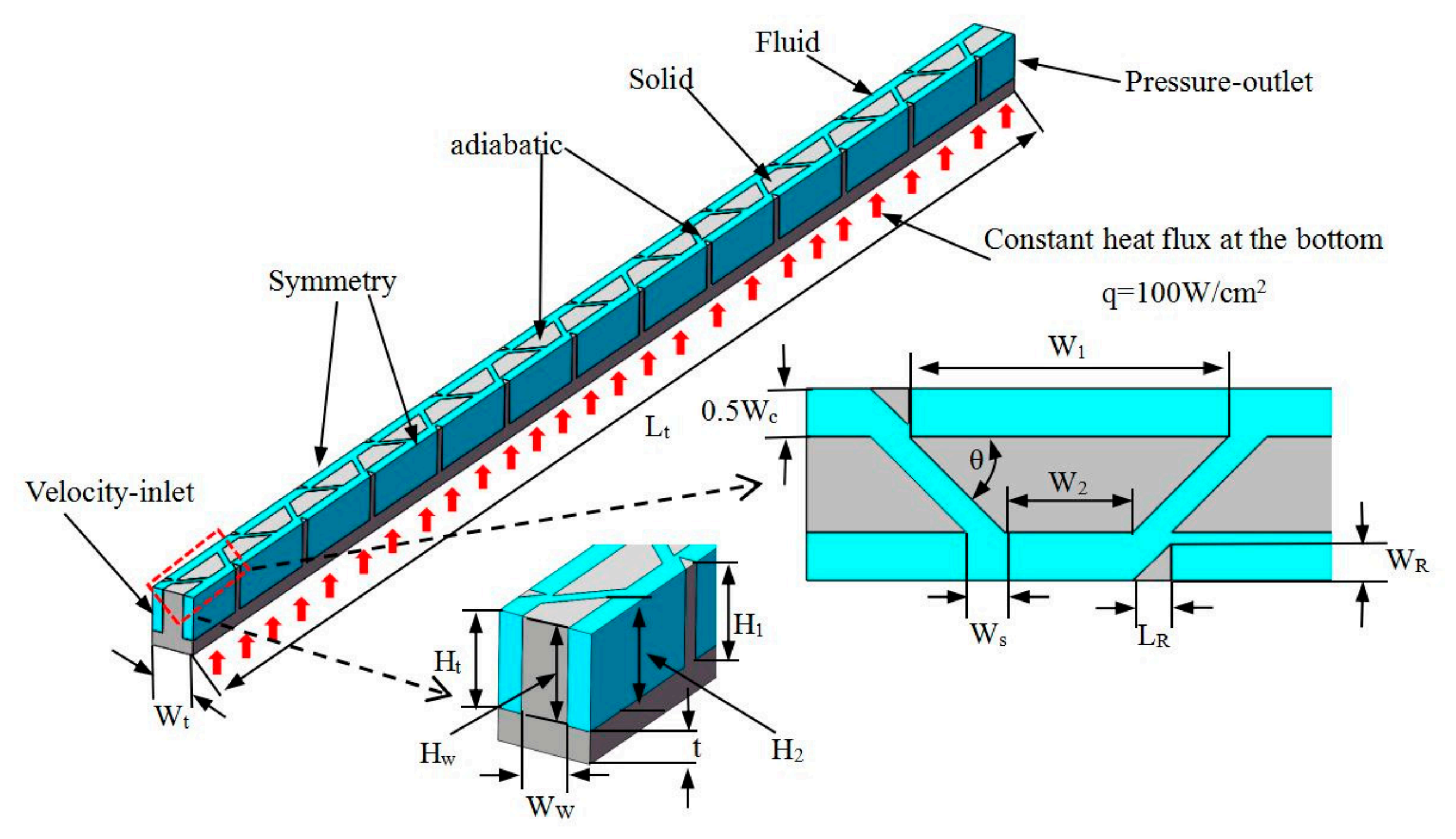
Schematic diagram of a single MCHS combined with composite front triangular ribs and secondary channels.

Figure 2 shows the computational physical domain and geometric dimensions of the novel MCHS with composite front triangular ribs and secondary channel structures. All key structural parameters adopted in the numerical model were explicitly defined and quantified as follows. The overall dimension parameters of the microchannel unit included a total width (*W*_t_) of 0.3 mm, a total length (*L*_t_) of 10 mm and a total height (*H*_t_) of 0.3 mm. In terms of the wall structure, the microchannel side wall width (*W*_w_) and height (*H*_w_) were 0.15 mm and 0.3 mm, and the structural thickness (*t*) of the microchannel was set to 0.1 mm. For the flow channel configuration, the main channel width (*W*_c_) and secondary channel width (*W*_s_) were 0.15 mm and 0.09 mm, respectively. Regarding the front triangular rib structure, its length (*L*_R_) and width (*W*_R_) were specified as 0.06 mm and 0.05 mm. For the interval structure of the secondary channel, the lower boundary length (*W*_1_) and upper boundary length (*W*_2_) were 0.44 mm and 0.14 mm, and the secondary channel formed a fixed inclination angle (*θ*) of 45° with the lower boundary of the interval [19]. Notably, the height of the front triangular ribs (*H*_1_) was 0.3 mm, and the height of the secondary channels (*H*_2_) was 0.3 mm.

### 2.2. Assumptions and Governing Equations

The MCHS investigated in this study is made of copper, with pure water serving as the coolant. Its material properties are detailed in Table 1. Several reasonable simplifying assumptions were adopted to reduce the complexity of numerical simulation, which are consistent with the basic simplification principles adopted in previous thermal-hydraulic simulations [19]:

(a) The cooling medium is in a single-phase laminar flow state with incompressible physical properties.
(b) Thermal physical properties of the solid heat sink material remain unchanged within the studied temperature range.
(c) All flow and heat transfer processes were treated as steady-state.
(d) For the established numerical model, thermal radiation from the heat sink outer walls and natural convective heat transfer with ambient air were neglected.

Based on the above simplifying assumptions, the governing equations were formulated to describe the fluid flow and heat transfer in the MCHS [19]. The continuity, momentum, and energy equations for the fluid domain and the energy equation for the solid domain were presented in Equations (1)–(4).

The complete mathematical expressions of the governing equations were listed as Equations (1)–(4).

The continuity equation for incompressible fluid flow is given by:

(1) $$\nabla \cdot \left(\rho_{f}\vec{V}\right)\;=\;0$$

The momentum equation describing the hydrodynamic motion of the working fluid is given by:

(2) $$\vec{V\cdot }\nabla (\rho_{f}\vec{V})=\;-\nabla P\;+\;\nabla (\mu_{f}\nabla \vec{V})$$

The corresponding energy equation is given by:

(3) $$\vec{V}\cdot \nabla (\rho_{f}C_{p,f}T_{f})\;=\;\nabla \;(\lambda_{f}\nabla T_{f})$$

The energy equation for the solid domain was simplified as:

*λ*_s_∇^2^*T*_s_ = 0 (4)

where *V* stands for flow velocity, *T* denotes medium temperature and *P* represents fluid pressure. *ρ* and *μ* separately refer to fluid density and dynamic viscosity. *C_p,f_* and *λ* correspond to constant pressure specific heat and thermal conductivity coefficient. Subscripts *f* and *s* are used to distinguish parameters of liquid cooling medium and solid substrate.

### 2.3. Boundary Condition Settings

Specific boundary conditions adopted in the numerical model were listed as follows [19]:

(1) Uniform velocity inlet was adopted for the channel inlet, with five inlet velocities (0.4, 0.8, 1.2, 1.6, 2.0 m/s) for comparative analysis.
(2) A constant temperature boundary condition of 300 K was specified at the coolant inlet.
(3) The pressure at the channel outlet was set to 0 Pa, *P* = *P*_out_ = 0 Pa.
(4) Temperature continuity is imposed at all contact interfaces between the solid substrate and the fluid domain, *T*_s_ = *T*_f_.
(5) Uniform constant heat flux load of 100 W/cm^2^ is loaded on the bottom heating surface of the MCHS.

### 2.4. Numerical Simulation and Data Processing

All numerical simulations in this study were carried out using the commercial CFD software Ansys Fluent R21. The finite volume method was adopted to discretize the governing partial differential equations. The SIMPLE algorithm was adopted for pressure–velocity coupling. A second-order upwind scheme was employed to discretize convective and diffusive terms for improved computational accuracy. For iterative convergence control, the residual limit for the continuity equation was set to less than 10^−6^, whereas a stricter criterion of 10^−9^ was specified for the energy equation to achieve converged steady-state heat transfer results.

To comprehensively assess the thermal and hydraulic performance of the newly designed MCHS, several key performance metrics were adopted in this work, including the Reynolds number (*Re*), Nusselt number (*Nu*), thermal resistance (*R_t_*), pumping power (*P_p_*), friction coefficient (*f*), pressure drop (*∆P*), and performance evaluation criterion (*PEC*).

The Reynolds number (*Re*) is introduced to characterize the flow state of coolant, and its specific calculation expression is shown in Equation (5).

(5) $$Re=\frac{\rho_{f}V_{in}D_{h}}{\mu_{f}}$$

where *V_in_* represents the average flow velocity at channel inlet, and *D_h_* is the hydraulic diameter of microchannel cross-section.

(6) $$D_{h}=\frac{2H_{t}W_{c}}{H_{t}+W_{c}}$$

The average Nusselt number (*Nu*) is calculated using the formula given below:

(7) $$Nu=\frac{qA_{s}D_{h}}{{\lambda_{f}NA}_{ch}(T_{s}-T_{f})}$$

where *T_s_*, *T_f_*, *A_s_*, *A_ch_*, q and *λ_f_* denote the average temperature of the microchannel wall, the average temperature of the fluid, the bottom heating area, the solid–liquid contact area, the effective heat flux, the thermal conductivity of fluid, respectively.

The thermal resistance (*R_t_*) is calculated using the formula given as follows:

(8) $$R_{t}=\frac{T_{s}-T_{f,in}}{qA_{s}}$$

The pumping power (*P_p_*) is calculated using the formula given below:

(9) $$P_{p}=\frac{N\dot{m}\Delta P}{\rho_{f}}$$

where $\dot{m}$ represents the mass flow rate of the fluid, and *N* is the total number of microchannels.

The average friction coefficient (*f*) can be obtained through the following formula:

(10) $$f=\frac{2∆pD_{h}}{{{\rho_{f}LV}_{in}}^{2}}$$

where *L* refers to the length of the channel and ∆p represents the pressure drop. The pressure drop is defined as follows:

(11) $$∆P=P_{in}-P_{out}$$

To comprehensively evaluate the trade-off between heat transfer enhancement and flow resistance increment, the *PEC* comprehensive performance evaluation index widely used in microchannel cooling research is adopted as the core evaluation criterion [20]. The mathematical expression of *PEC* is given in Equation (12), which takes both relative Nusselt number and relative friction coefficient into consideration.

(12) $$PEC=\frac{\left(\frac{Nu}{Nu_{s}}\right)}{({\frac{f}{f_{s}})}^{1/3}}$$

where *Nu* and *f* denote the Nusselt number and friction factor corresponding to the modified open MCHS configurations (i.e., MC-OSC, MC-OFTR, MC-OSOC, MC-FTROSOC, MC-OFTRSOC and MC-OFTROSOC). By contrast, *Nu_s_* and *f_s_* denote the two parameters of the conventional smooth closed MCHS (i.e., MC-SC) [20].

### 2.5. Grid Independence Verification

The MCHS with composite front triangular ribs and secondary channels was meshed using tetrahedral elements. A grid independence test was performed to eliminate numerical errors induced by grid density, as shown in Table 2. Six mesh configurations with different refinement levels were generated in this work, and the corresponding numbers of grid cells were 50,909, 80,835, 159,954, 367,273, 1,252,007 and 4,268,000, respectively. Comparative results showed that when the number of grid cells reached 367,273, the relative deviations in the maximum substrate temperature and channel pressure drop were only 0.044% and 0.013% compared with the ultra-fine grid scheme. Considering both computational efficiency and numerical accuracy, the mesh scheme containing 367,273 grid cells was selected for all subsequent comparative simulation cases.

### 2.6. Computational Model Verification

**Figure 3:**
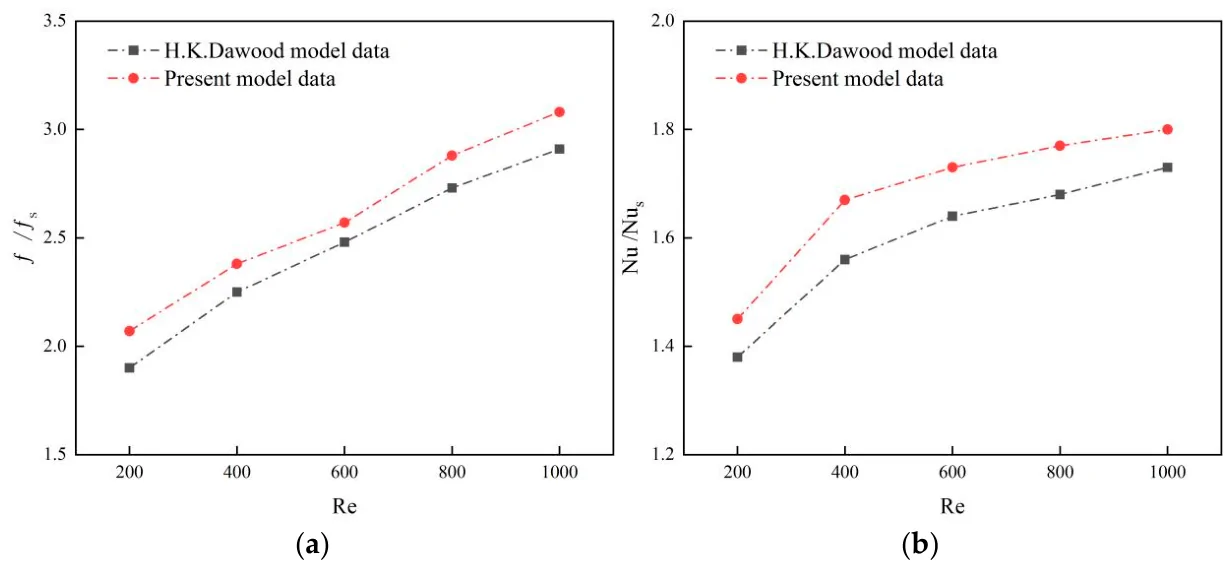
Comparison between the simulation results from H. K. Dawood [20] and the present simulated results: (**a**) *ƒ/ƒ*_s_ and (**b**) *Nu/Nu*_s_ under different Reynolds numbers.

To verify the reliability of the numerical simulation constructed in this study, the thermal-hydraulic results calculated by the present model were compared with the simulation results by Dawood et al. [20]. The comparisons are shown in Figure 3a,b. Comparison curves of normalized friction coefficient and Nusselt number at various Reynolds numbers revealed that the data variation trend of this work was in good agreement with the reported data, with average relative errors of approximately 5.5% for *ƒ*/*ƒ*_s_ and 5.2% for *Nu*/*Nu*_s_. All deviations fall within an acceptable range for numerical simulations of microchannel heat sinks. These comparisons demonstrated that the model parameter settings and solution strategies adopted in this work were reliable and can provide support for subsequent structural analyses.

### 2.7. Performance Comparison of Different Schemes

**Figure 4:**
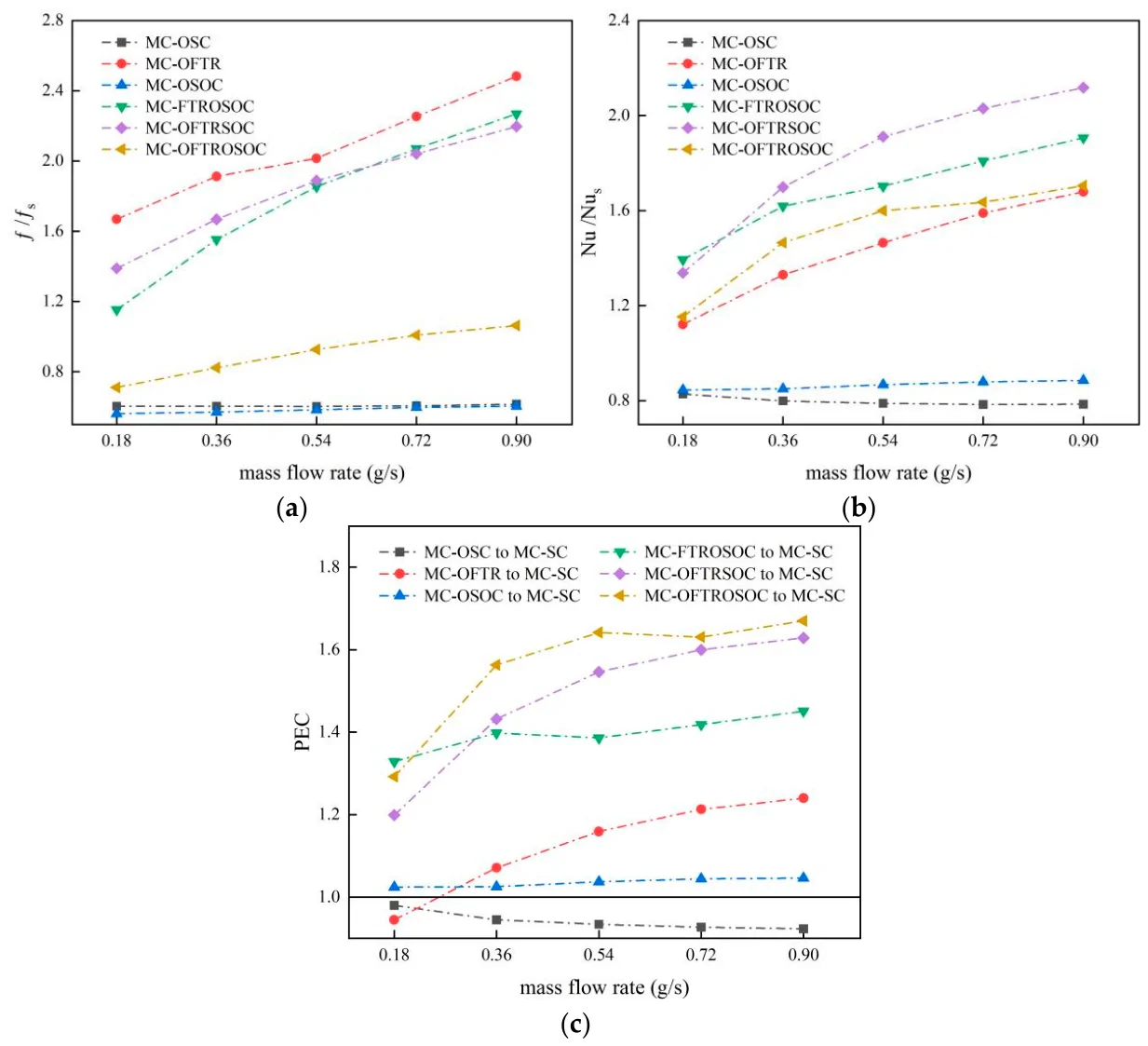
Performance comparison between the proposed microchannels (MC-OSC, MC-OFTR, MC-OSOC, MC-FTROSOC, MC-OFTRSOC, MC-OFTROSOC) and closed straight rectangular microchannel (MC-SC): (**a**) *ƒ/ƒ_s_*; (**b**) *Nu/Nu_s_*; (**c**) *PEC*.

To investigate how various geometric configurations affect the thermal-hydraulic performance and cooling capacity of MCHS, seven different structural designs were proposed for comparative numerical analysis in this work. Figure 4 compares the performance parameters including the friction factor ratio (*ƒ/ƒ_s_*), Nusselt number ratio (*Nu/Nu_s_*), and *PEC* of open microchannels (MC-OSC, MC-OFTR, MC-OSOC, MC-FTROSOC, MC-OFTRSOC and MC-OFTROSOC) and closed straight rectangular microchannels (MC-SC) under various mass flow rates. The variation curves of friction factor ratio (*ƒ/ƒ_s_*) against different mass flow rates for multiple microchannel configurations are plotted in Figure 4a. Elevated friction factor values indicated that the fluid experienced greater flow resistance inside the channel. As can be seen from Figure 4a, the friction coefficients of MC-OFTR, MC-FTROSOC and MC-OFTRSOC were relatively high. This can be explained by the fact that the front triangular ribs increased the resistance when the fluid flowed through the channel, thereby producing a larger pressure drop. MC-OFTR exhibited the highest friction factor. This was attributed to the fluid impinging on the open-front triangular ribs and flowing through closed straight rectangular microchannels, which markedly increased the friction factor. In addition, MC-OSC, MC-OSOC and MC-OFTROSOC exhibited lower friction factors. Such behavior stemmed from the fact that they all adopted fully open microchannels with secondary flow channels, thereby requiring a smaller pressure drop to maintain the flow. Figure 4b presents the normalized average Nusselt number ratio (*Nu/Nu_s_*) under various mass flow rates for different MCHS structures. A higher average Nusselt number corresponds to intensified convective heat transfer between fluid and solid walls, signifying enhanced heat transfer performance. As can be seen from Figure 4b, MC-OFTR, MC-FTROSOC, MC-OFTRSOC and MC-OFTROSOC had excellent heat transfer performance. This was due to the design of the front triangular ribs and open secondary channels in the microchannels, which increased the contact area with the coolant and effectively enhanced the heat dissipation capacity. Figure 4c illustrates the influence of different geometric configurations on the *PEC* at various mass flow rates. A higher *PEC* value indicates superior overall performance of the MCHS. As can be seen from Figure 4c, the *PEC* values of the MC-FTROSOC, MC-OFTRSOC and MC-OFTROSOC were always greater than 1, which indicated that the overall performance of these three schemes was better than that of the traditional MC-SC. When the mass flow rate was 0.9 g/s, the comprehensive heat transfer performance of MC-OFTROSOC was the best, with a *PEC* of 1.67. The research results showed that the comprehensive heat transfer performance of the MC-OFTROSOC was superior to that of other design schemes. This was because microchannels with front triangular ribs introduced additional flow resistance and pressure drop, while the open top structure of the channel effectively reduced flow resistance. The front triangular ribs and secondary channels enlarged the fluid–solid contact area and enhanced heat transfer. The combination of open front triangular ribs and open secondary channels achieved a trade-off between thermal and hydraulic characteristics, thereby maximizing the PEC value.

**Figure 5:**
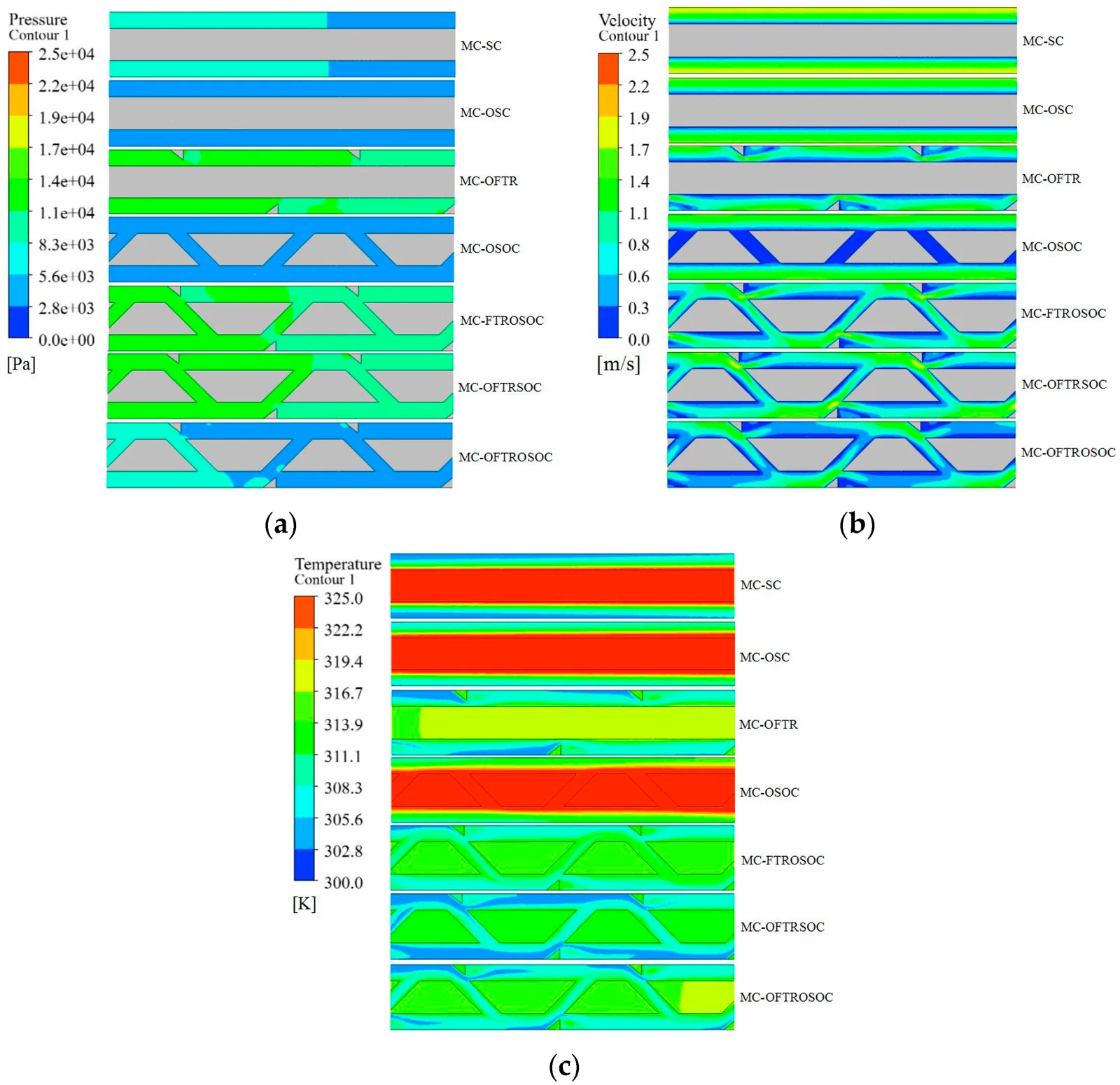
(**a**) pressure (**b**) velocity (**c**) temperature distribution contours at contrastive structure in mass flow rate = 0.54 g/s for x-z plane with *y* = 0.1 mm.

At a mass flow rate of 0.54 g/s, Figure 5a–c show pressure, velocity, and temperature contours extracted from the central x-z cross-section (*y* = 0.1 mm) of MCHS with various geometric designs. As can be seen from Figure 5a, the pressure drop of the MCHS with front triangular ribs was higher than that of the traditional closed straight rectangular microchannel and the microchannel with open secondary channels. Compared with MC-FTROSOC and MC-OFTRSOC, the MC-OFTROSOC exhibited a lower pressure drop. The widened flow area provided by its open front triangular ribs and open secondary channels reduced flow resistance, which consequently decreased the pressure drop within the heat sink. As shown in Figure 5b,c, MC-SC, MC-OSC and MC-OSOC exhibited higher overall temperature compared with other configurations. This was because the above mentioned microchannels were not equipped with structures for active flow perturbation and thermal boundary-layer disruption. In MC-SC and MC-OSC, the fluid exhibited highly ordered parallel streamlines. The thermal boundary layer continuously developed and thickened along the flow direction, substantially reducing the near-wall heat transfer performance. Although MC-OSOC incorporated open secondary channels, it lacked the impinging disturbance induced by ribs. Its flow field remained relatively stable without intense vortex mixing; thus, the thermal boundary layer could hardly be effectively disrupted, resulting in limited heat transfer enhancement. Furthermore, MC-SC, MC-OSC and MC-OSOC possessed markedly smaller effective convective heat transfer areas than other composite microchannel configurations, which further limited their heat dissipation capability under high heat flux operating conditions. For MC-OFTROSOC, distinct high-velocity impingement zones were formed upstream of the triangular ribs, while low-speed laminar recirculation vortices were generated behind the ribs. Such vortex structures strongly disrupted the thermal boundary layer and enhanced mixing between the near-wall high-temperature fluid and the low-temperature mainstream coolant, thereby significantly improving convective heat transfer performance. Compared with other geometric configurations, the composite structure integrating open front triangular ribs and open secondary channels significantly regulated and optimized the internal flow characteristics of MCHS. This was attributed to its larger effective heat transfer area and increased flow area. Meanwhile, the intensified laminar-flow-generated vortex strength generated by the open front triangular ribs and the elevated fluid velocity within the open secondary channels collectively enhanced the overall thermal-hydraulic performance.

## 3. Effects of Key Design Variables on Performance of MCHS

### 3.1. Effects of Open Front Triangular Ribs Heights

**Figure 6:**
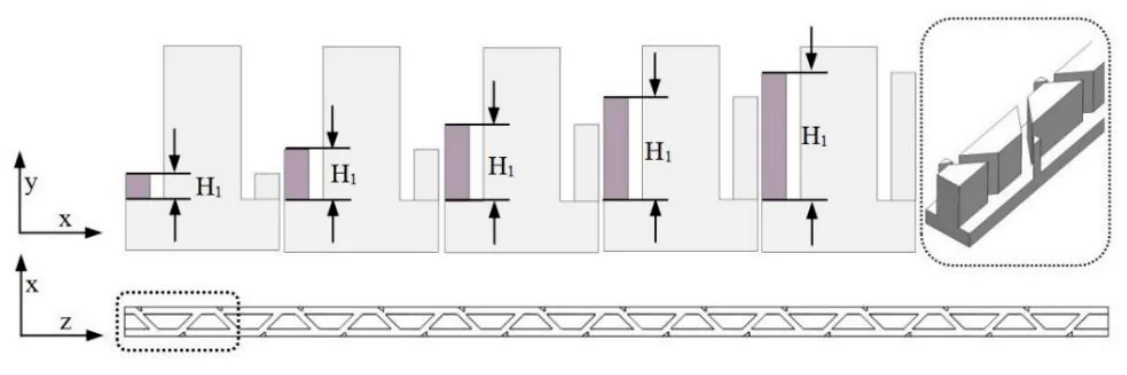
Side views of the heat sink for *H*_1_ = 0.05 mm, 0.10 mm, 0.15 mm, 0.20 mm and 0.25 mm.

The MC-OFTRSOC structure was selected as the research object to further explore the effects of varying open front triangular rib heights on the thermal-hydraulic performance of MCHS. Five different *H*_1_ values were selected for detailed parametric analysis, namely 0.05 mm, 0.10 mm, 0.15 mm, 0.20 mm and 0.25 mm, and the corresponding configurations were displayed in Figure 6.

**Figure 7:**
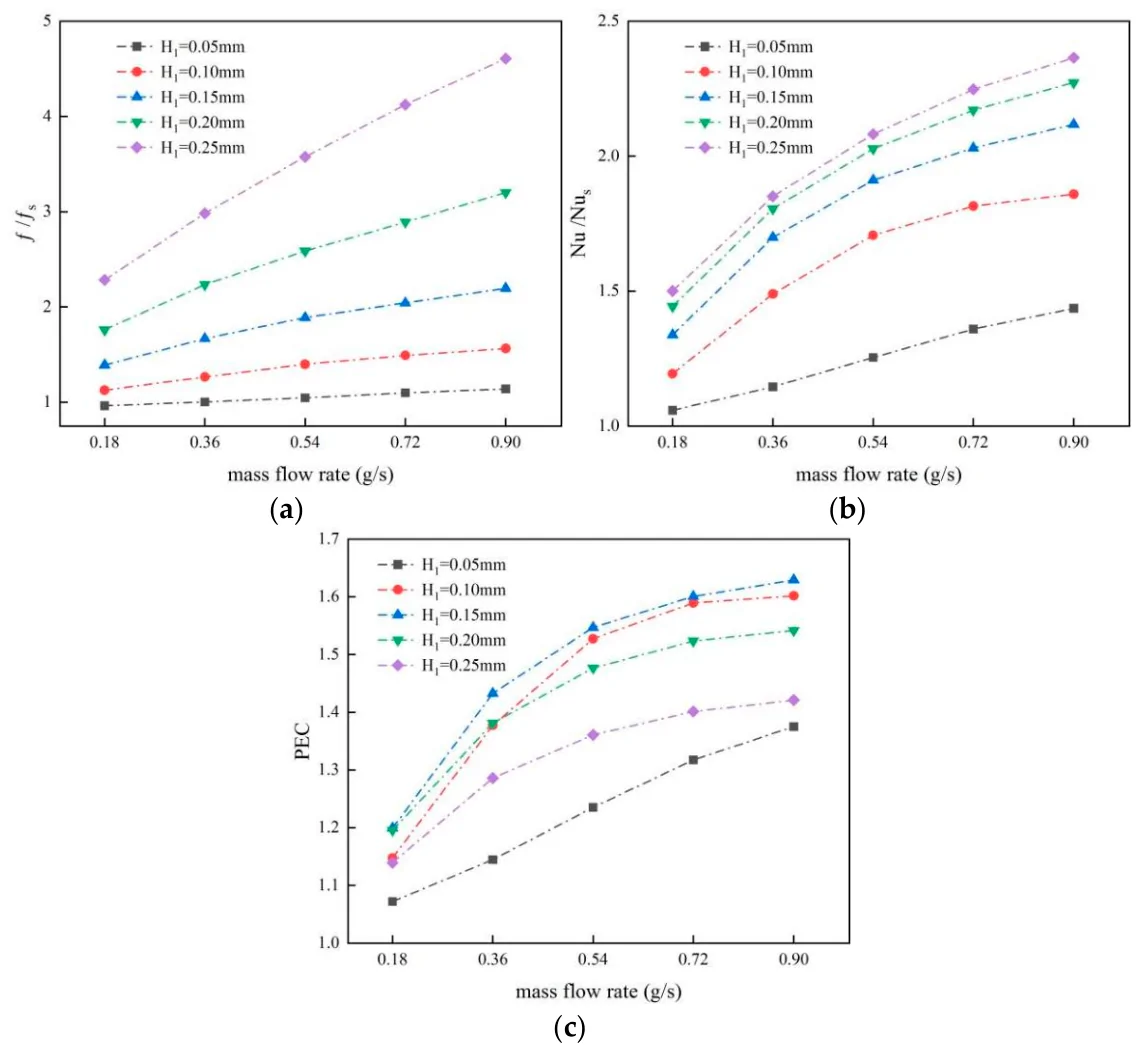
Variations of (**a**) *ƒ/ƒ_s_*, (**b**) *Nu/Nu_s_* and (**c**) *PEC* with rib height *H*_1_ under different mass flow rates.

Figure 7 illustrates the variations of *ƒ/ƒ_s_*, *Nu/Nu_s_* and *PEC* with *H*_1_ for the five designed microchannel schemes at different mass flow rates. Figure 7a shows the influence of H_1_ on the normalized friction factor at different mass flow rates. Within the investigated range of H_1_, *ƒ/ƒ_s_* increased with increasing mass flow rate and H_1_. This was because increasing the height of open front triangular ribs enhanced the flow resistance of the coolant, thereby raising the pressure drop and friction factor. Figure 7b shows the influence of *H*_1_ on *Nu/Nu_s_* at different mass flow rates. Within the investigated range of *H*_1_, *Nu/Nu_s_* increased with increasing mass flow rate and *H*_1_. This was attributed to the expanded net convective contact area between the coolant and the open front triangular ribs in the microchannels, which improved the heat transfer performance accordingly. Figure 7c illustrates the effect of *H*_1_ on *PEC* at different mass flow rates. The *PEC* values of all five structural schemes were consistently greater than 1 compared with the reference MC-SC, indicating that the comprehensive thermal-hydraulic performance of the five proposed structures was superior to that of MC-SC. Within the investigated range, *PEC* increased gradually with rising mass flow rate. At the mass flow condition of 0.9 g/s, the MC-OFTRSOC structure with *H*_1_ fixed at 0.15 mm delivered the maximum *PEC* value of 1.62. Such comparison results verified that the MCHS equipped with matched open front triangular ribs and secondary channels proposed in this paper could improve overall thermal-hydraulic performance.

### 3.2. Effect of Open Secondary Channels Heights

**Figure 8:**
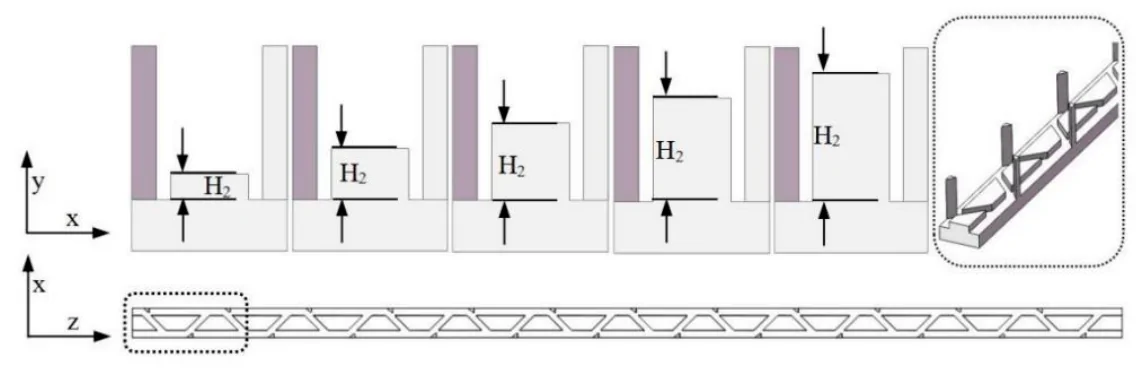
Side of the heat sink for *H*_2_ = 0.05 mm, 0.10 mm, 0.15 mm, 0.20 mm and 0.25 mm.

The MC-FTROSOC structure was selected as the research object to further explore the effects of varying open secondary channel heights on the thermal-hydraulic characteristics of MCHS. Five different *H*_2_ values were selected for detailed parametric analysis, namely 0.05 mm, 0.10 mm, 0.15 mm, 0.20 mm and 0.25 mm, and the corresponding configurations were displayed in Figure 8.

**Figure 9:**
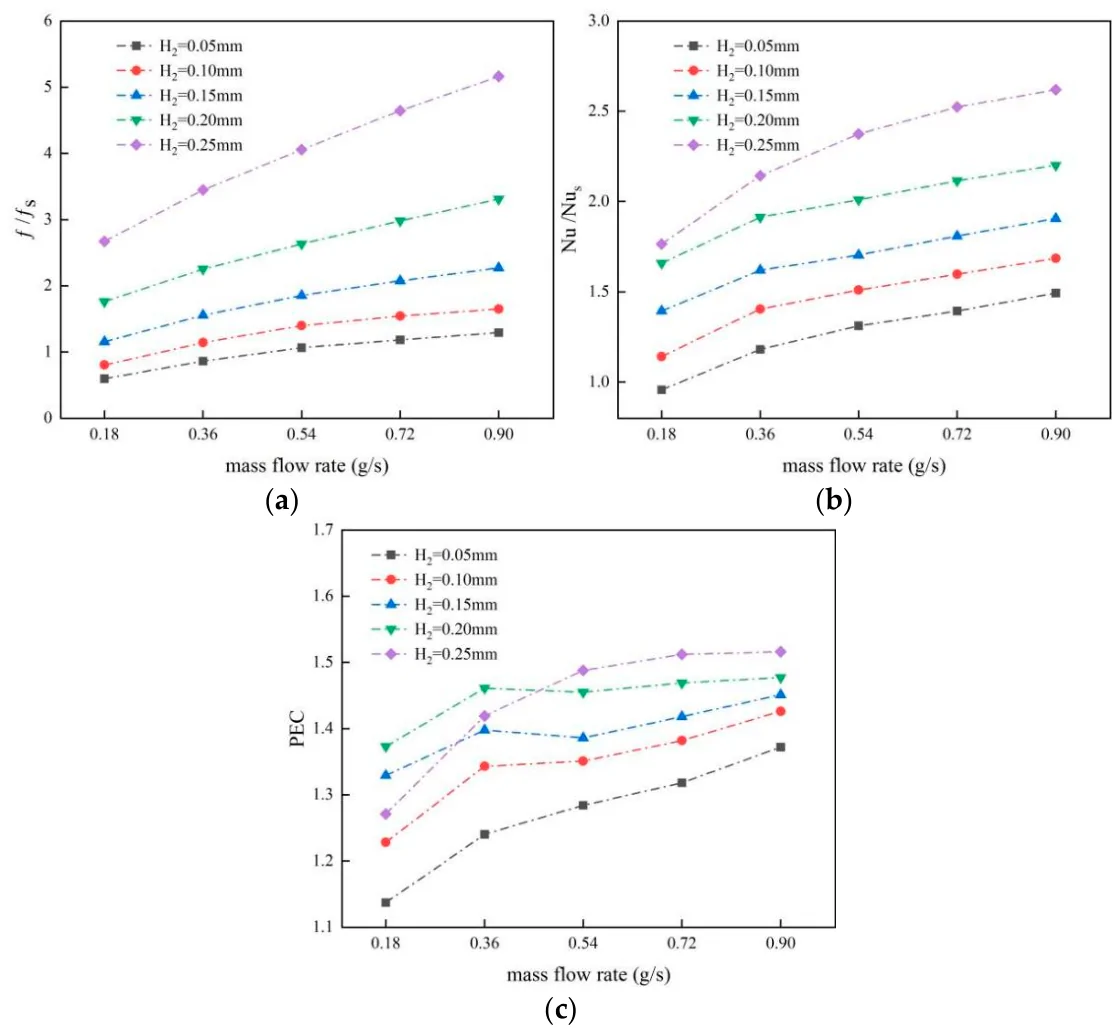
Variations of (**a**) *ƒ/ƒ_s_*, (**b**) *Nu/Nu_s_* and (**c**) *PEC* with *H*_2_ under different mass flow rates.

Figure 9 illustrates the variations of *ƒ/ƒ_s_*, *Nu/Nu_s_* and *PEC* with *H*_2_ for the five designed microchannel schemes at different mass flow rates. Figure 9a shows the effect of *H*_2_ on the normalized friction factor at different mass flow rates. Within the studied range of open secondary channel heights, *ƒ/ƒ_s_* gradually rose with increases in mass flow rate and *H*_2_. This was attributed to the increased flow resistance encountered by the coolant as the height of open secondary channels increased, which raised pressure drop and friction factor. Figure 9b shows the influence of *H*_2_ on *Nu/Nu_s_* at different mass flow rates. Within the investigated range of *H*_2_, *Nu/Nu_s_* increased gradually with the rise in mass flow rate and grew with increasing *H*_2_. This was because open secondary channels provided the enlarged net convective surface area, thereby effectively improving the overall heat transfer performance. Figure 9c illustrates the effect of *H*_2_ on *PEC* at different mass flow rates. The *PEC* values of all five structural schemes were consistently greater than 1, indicating that the proposed structures yielded better comprehensive thermal-hydraulic performance than the reference MC-SC. Within the investigated range, *PEC* increased gradually with rising mass flow rate and finally stabilized. At the mass flow condition of 0.9 g/s, the MC-FTROSOC structure with *H*_2_ fixed at 0.25 mm delivered the maximum *PEC* value of 1.51. These results demonstrated that the MCHS equipped with matched front triangular ribs and open secondary channels proposed in this paper could improve overall thermal-hydraulic performance.

## 4. Optimization of the Composite Open MCHS

### 4.1. Optimization Variables and Objective Function

To obtain the optimal structure of the MCHS with composite open front triangular ribs and open secondary channels that delivered superior heat transfer performance, orthogonal experimental design was employed to generate 25 test groups with three-factor and five-level. A constant inlet mass flow rate of 0.54 g/s was adopted for all orthogonal cases. *H*_1_, *H*_2_ and *W*_w_ were selected as the design variables, and the *PEC* was defined as the optimization objective. The ranges of *H*_1_, *H*_2_ and *W_w_* were 0.06–0.3 mm, 0.06–0.3 mm and 0.09–0.17 mm, respectively. The orthogonal experimental design and the *PEC* values calculated by CFD were presented in Table 3 and Table 4.

### 4.2. Construction of Surrogate Model for Objective Function

Based on the data in Table 4, a polynomial function of *PEC* was fitted via the second-order response surface model, and a surrogate model for *PEC* was constructed. Specifically, the 24th group was deemed potentially suspicious and excluded from the fitting procedure. The response function was defined by a second-order polynomial consisting of intercept, linear, pure-quadratic and two-factor interaction terms, as shown below:

(13) $$y(x)=\beta_{0}+\sum_{i=1}^{k}\beta_{i}x_{i}+\sum_{i=1}^{k}\beta_{ii}x_{i}^{2}+\sum_{i<j}^{k}\beta_{ij}x_{i}x_{j}$$

where $x_{i}$ denotes each design variable, namely *H*_1_, *H*_2_ and *W*_w_, *k* stands for total number of design variables, and β represents the model coefficient.

In this paper, the surrogate model for *PEC* was constructed using multiple linear regression in MATLAB R2016b. For our experimental dataset, the quadratic term *W*_w_^2^ exhibited collinearity with other model terms, which rendered the design matrix rank-deficient. Consequently, this term could not be reliably estimated and was excluded from the fitted model, as follows:

(14) $$\begin{array}{l} PEC=-0.058801+13.707*H_{1}+5.4095*H_{2}+3.3282*W_{w}-26.401*{H_{1}}^{2}\\ -6.2529*{H_{2}}^{2}-6.3445*H_{1}*H_{2}-18.216*H_{1}*W_{w}-14.674*H_{2}*W_{w} \end{array}$$

To assess the predictive precision of the surrogate model, the coefficient of determination (*R^2^*), adjusted coefficient of determination ($R_{adj}^{2}$), sum of squared errors (SSE) and root mean square error (*RMSE*) were adopted in this study, and the corresponding formulas were presented as follows [35].

(15) $$R^{2}=1-\frac{\sum_{i=1}^{m}(h_{i}-{\bar{y}}_{i}{)}^{2}}{\sum_{i=1}^{m}(h_{i}-\bar{h}{)}^{2}}$$

(16) $$RMSE=\sqrt{\frac{1}{m}\sum_{i=1}^{m}(h_{i}-y_{i}{)}^{2}}$$

(17) $$SSE=\sum_{i=1}^{m}(h_{i}-y_{i}{)}^{2}$$

(18) $$R_{adj}^{2}=1-\frac{\frac{1}{m-p-1}\sum_{i=1}^{m}{\left(h_{i}-y_{i}\right)}^{2}}{\frac{1}{m-1}\sum_{i=1}^{m}{\left(h_{i}-\bar{h}\right)}^{2}}$$

where *m* is the number of samples, *h*_i_ is the experimental value, *y*_i_ is the predicted value, *p* is the number of independent variables of the model and $\bar{h}$ is the average of experimental values. The closer *R*^2^ is to 1, the higher the fitting accuracy of the model equation.

**Figure 10:**
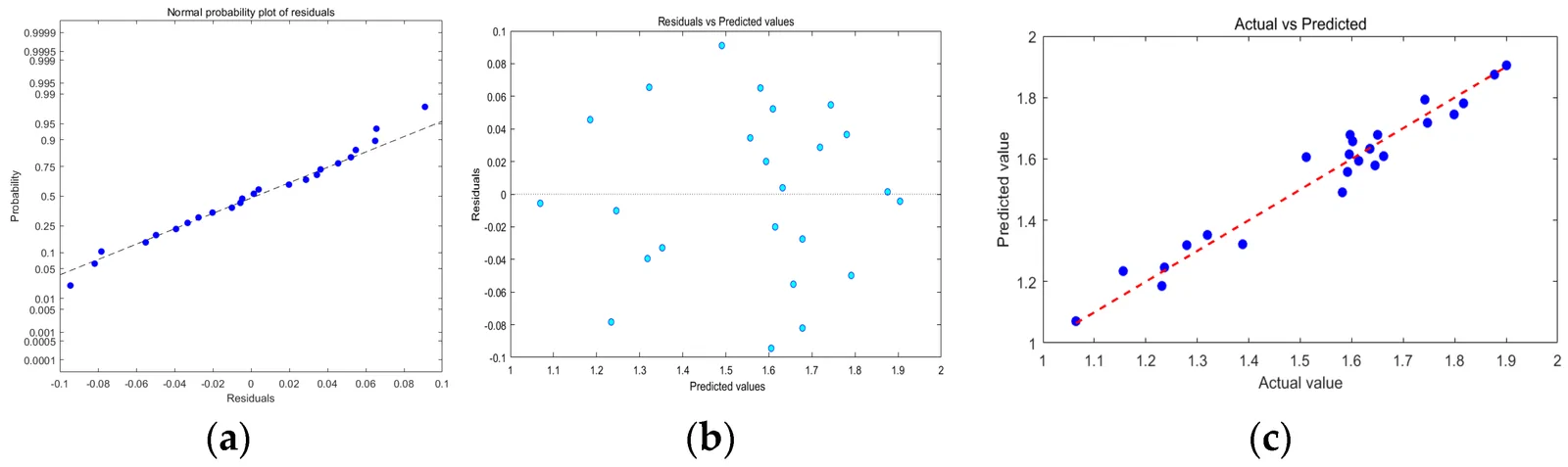
Residual analysis of the PEC surrogate model. (**a**) Normal probability plot of residuals. (**b**) Residuals and predicted values. (**c**) In sample fitting diagnostic: comparison between actual and predicted values.

The ANOVA for the *PEC* surrogate model revealed that the regression model was significant (*F* = 37.1, *p* < 0.0001). For the *PEC* surrogate model, the values of *R*^2^, adjusted *R*^2^, *RMSE* and *SSE* were 0.952, 0.926, 0.063 and 0.0596, respectively. The *R*^2^ and $R_{adj}^{2}$ were close to 1, while the *RMSE* was close to 0, indicating that the fitted curve matched the numerical results well with high fitting accuracy. Residual analysis of the *PEC* surrogate model is shown in Figure 10. The normal probability plot of residuals in Figure 10a shows that the residuals approximately followed a normal distribution. As shown in Figure 10b, the residuals were randomly scattered between −0.1 and 0.1, without obvious curvature, funnel-shaped pattern, or systematic trend. Figure 10c presents the in-sample fitting diagnostic comparison between actual and predicted values. Most data points distributed closely along the ideal diagonal line, which indicated a satisfactory goodness of fit of the present surrogate model on the training dataset. This indicated that the model satisfied the homoscedasticity assumption, and no significant systematic bias existed. Accordingly, this fitted equation could be used for subsequent optimization of structural parameters.

### 4.3. Global Parametric Optimization Based on Genetic Algorithm

In this study, the genetic algorithm was adopted for parametric optimization of the composite MCHS with open front triangular ribs and open secondary channels. The mathematical model for the optimization problem was formulated as follows.

(19) $$\{\begin{array}{c} \max f\left(H_{1},H_{2},W_{w}\right)=\max\left\{PEC\left(H_{1},H_{2},W_{w}\right)\right\}\\ s.t.\;0.06\;\mathrm{mm}\leq H_{1}\leq 0.3\;\mathrm{mm}\\ \;0.06\;\mathrm{mm}\leq H_{2}\leq 0.3\;\mathrm{mm}\\ \;0.09\;\mathrm{mm}\leq W_{w}\leq 0.17\;\mathrm{mm} \end{array}$$

**Figure 11:**
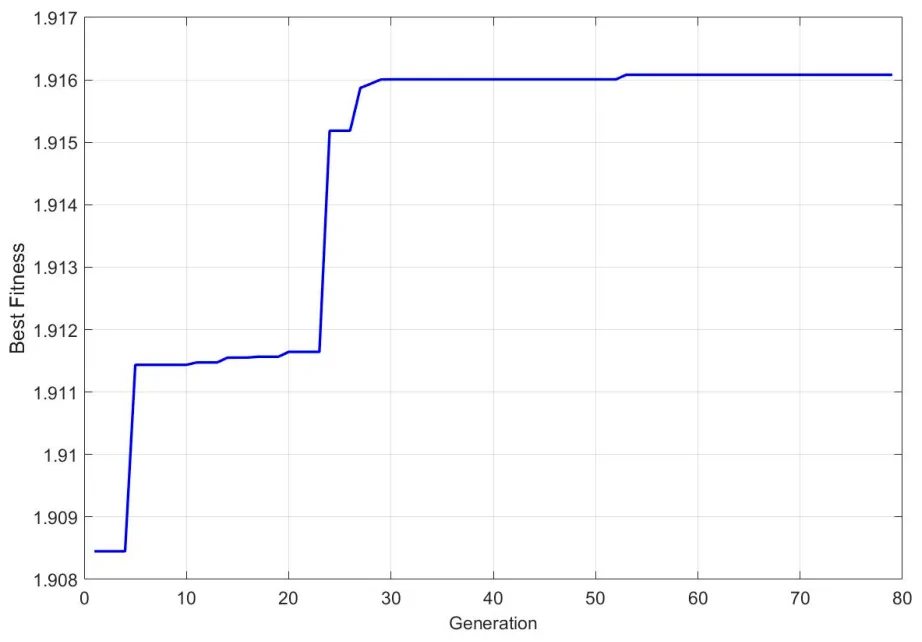
Fitness curve of global optimization by GA.

For the genetic algorithm adopted in this work, the population size was set to 100 with a maximum of 200 iterations. Meanwhile, the crossover probability was fixed at 0.8 and the mutation probability was set to 0.1. The optimization objective was to maximize the overall performance coefficient (*PEC*) of the MCHS. After 200 iterations, the overall performance coefficient optimized globally by the genetic algorithm converged to 1.916, as shown in Figure 11. 

### 4.4. Optimization Result Verification & Comparative Analysis

The genetic algorithm (GA) was adopted to optimize the structural parameters of the novel MCHS with open front triangular ribs and open secondary channels to obtain optimal design configurations. Numerical simulation verifications were carried out, and the corresponding results were listed in Table 5. Table 5 shows that the optimal structural parameters with *H*_1_ = 0.20 mm, *H*_2_ = 0.22 mm and *W*_w_ = 0.09 mm yielded *PEC* values of 1.916 and 1.917 from genetic algorithm prediction and numerical simulation verification, respectively, corresponding to a low relative error of 0.05%. The numerical simulation results exhibited good agreement with the GA predicted values. This validated that the genetic algorithm was capable of efficiently identifying the optimal structural parameters of the MCHS with superior overall heat transfer performance.

The comparison and verification results of the CFD simulations are presented in Table 6. This table compares three cases: the original design, the optimized design, and the design reported in Ref. [19]. Compared with the original design, the optimized MCHS reduced the average inlet-outlet pressure drop by 12.6%, thermal resistance by 8.5%, and pumping power by 18.2%, while the *PEC* increased by 11.5%. These improvements fulfilled the optimization objective of enhancing the overall thermal-hydraulic performance while reducing the flow pressure drop. Compared with the design reported in Ref. [19], the optimized MCHS reduced the average inlet-outlet pressure drop by 68.9%, thermal resistance by 15.3%, and pumping power by 70.9%, while the *PEC* increased by 55.7%. It was demonstrated that the proposed composite open microchannel structure optimized using the genetic algorithm effectively balanced heat transfer enhancement and flow resistance reduction, leading to a substantial improvement in overall thermal-hydraulic performance.

**Figure 12:**
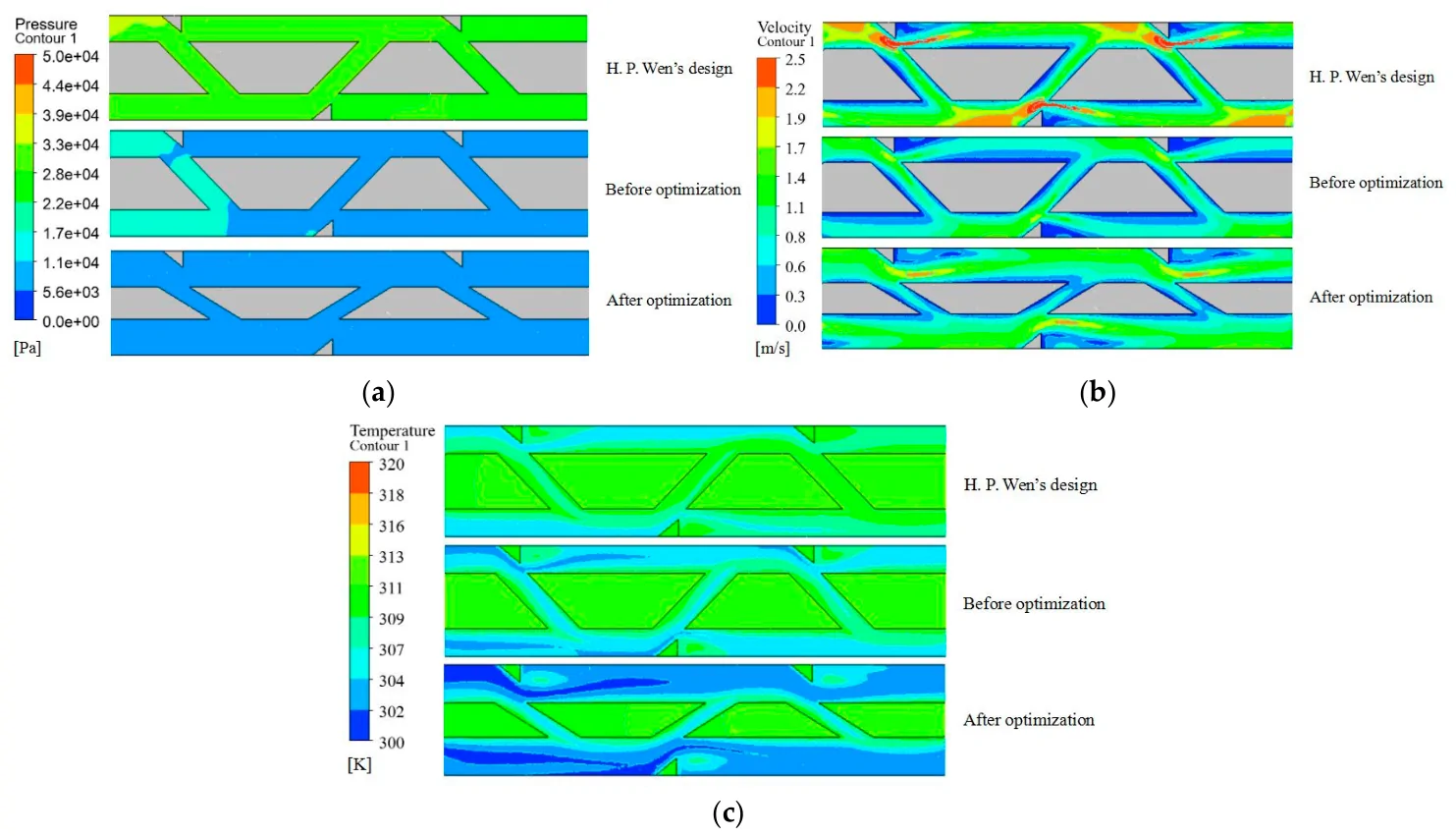
(**a**) Pressure distribution contour, (**b**) velocity distribution profile and (**c**) temperature distribution contour in the x-z plane at y = 0.1 mm and mass flow rate of 0.54 g/s, for pre-optimization, post-optimization and H. P. Wen’s design.

Figure 12 illustrates the pressure, velocity and temperature contours on the x-z plane at y = 0.1 mm for the original design, the optimized design and the reference design from Ref. [19], under a mass flow rate of 0.54 g/s. The results show that the total pressure drop of the optimized design was reduced by 12.6% and 68.9%, compared with the original design and the configuration in Ref. [19], respectively. Such performance improvement could be attributed to the structural advantages of the optimized geometry, which effectively enlarged the flow passage area of the main channel and expanded the open flow region at the top of the channel. This structure substantially weakened the flow-blocking effect of the coolant and accordingly reduced the overall flow resistance and pressure drop. Compared with the reference design in Ref. [19], the optimized design provided a larger open space at the channel top and yielded a lower overall flow velocity under a constant mass flow rate. Compared with the original design, the local velocity within the main channel of the optimized design was obviously higher than that of the original design. Meanwhile, multiple laminar-flow-generated vortices were formed behind the open front triangular ribs. These vortex structures disrupted the developing thermal boundary layer and enhanced the mixing between high temperature and low temperature coolant streams. Compared with the original design and the design in Ref. [19], the maximum temperature of the optimized microchannel heat sink was reduced by 1.5 K and 2.9 K, respectively, accompanied by an improvement in temperature uniformity. It could be concluded that global optimization based on GA could be effectively adopted for the design of composite open MCHSs to obtain the structural parameters with optimal overall performance.

## 5. Conclusions

To enhance the overall thermal-hydraulic performance of MCHSs, six novel open microchannel structures with different geometric configurations were proposed in this paper, and a systematic comparative analysis of their flow and heat transfer characteristics was carried out. Numerical results demonstrated that the open front triangular ribs acted as critical flow guiding structures, which effectively guided the coolant into the secondary channels. Meanwhile, the enlarged flow area provided by the open secondary channels could mitigate the unfavorable pressure drop increment stemming from the front triangular ribs. Parametric analyses were also performed to investigate the flow and heat transfer characteristics of the MCHS with different geometric parameters *H*_1_ and *H*_2_. The genetic algorithm was used for the global optimization of the open MCHS to obtain the optimal combination of structural parameters that reduced the total pressure drop and improved the comprehensive thermal-hydraulic performance simultaneously. The main conclusions of this study are summarized as follows:

(1) Compared with the other six schemes, the composite MCHS with open front triangular ribs and open secondary channels proposed in this work (MC-OFTROSOC) provides a larger effective convective heat transfer area and sufficient open flow space, and exhibits the optimal overall thermal-hydraulic performance.
(2) Single-factor analyses were carried out to investigate the effects of *H*_1_ and *H*_2_ on the thermal-hydraulic performance of the MCHS. When either *H*_1_ or *H*_2_ was increased separately, both the friction factor ratio *ƒ*/*ƒ*_s_ and the Nusselt number ratio *Nu*/*Nu*_s_ increased with the corresponding parameter. When *H*_1_ = 0.25 mm, the maximum *Nu*/*Nu*_s_ value obtained was 2.36, and when *H*_2_ = 0.25 mm, the maximum *Nu*/*Nu*_s_ value obtained was 2.61. When *H*_1_ = 0.25 mm, the maximum *f*/*f* _s_ value was 4.60. When *H*_2_ = 0.25 mm, the maximum *f*/*f* _s_ value was 5.16. At a mass flow rate of 0.9 g/s, the MC-OFTRSOC with *H*_1_ = 0.15 mm exhibited the best comprehensive heat transfer performance with a *PEC* of 1.62. At the same mass flow rate, the MC-FTROSOC with *H*_2_ = 0.25 mm achieved the best comprehensive heat transfer performance with a *PEC* value of 1.51.
(3) Global parameter optimization of the open MCHS was conducted using a genetic algorithm. The optimal structural parameters were determined as *H*_1_ = 0.20 mm, *H*_2_ = 0.22 mm, and *W*_w_ = 0.09 mm. Under this parameter combination, the open MCHS achieved the best comprehensive thermal-hydraulic performance with a *PEC* of 1.917, corresponding to the optimal pressure and temperature field distributions.
(4) Compared with the original design and the design reported in Ref. [19], the optimized open MCHS exhibits remarkable performance improvements. Its average inlet-outlet pressure drop, thermal resistance and pumping power are reduced by up to 68.9%, 15.3% and 70.9%, respectively, while the *PEC* is increased by a maximum of 55.7%.

## Figures and Tables

**Table 1:** Material properties.

Materials	*ρ*/(kg/m^3^)	*C_p_*/(J/(kg·K))	*λ*/(W/(m·K))
Cu	8940	385	400
H_2_O	998.2	4182	0.6

**Table 2:** Grid independence verification results.

Grid size (mm)	0.030	0.025	0.020	0.015	0.010	0.005
Grid number	50,909	80,835	159,954	367,273	1,252,007	4,268,000
∆P [Pa]	4553.659	4562.559	4569.859	4575.459	4576.659	4577.481
$T_{max}$ [K]	326.895	327.295	327.615	327.901	327.932	327.945
∆P error [%]	0.320%	0.198%	0.101%	0.013%	0.004%	criterion
$T_{max}$ error [%]	0.520%	0.326%	0.167%	0.044%	0.018%	criterion

**Table 3:** Levels of each factor.

Factor	1	2	3	4	5
Open front triangular rib heights (*H*_1_)	0.06	0.12	0.18	0.24	0.30
Open secondary channel heights (*H*_2_)	0.06	0.12	0.18	0.24	0.30
Microchannel sidewall widths (*W*_w_)	0.09	0.11	0.13	0.15	0.17

**Table 4:** Orthogonal experimental design and results.

Number	*H*_1_	*H*_2_	*W*_w_	*PEC*	Number	*H*_1_	*H*_2_	*W*_w_	*PEC*
1	0.06	0.06	0.09	1.064	14	0.18	0.24	0.09	1.900
2	0.06	0.12	0.11	1.156	15	0.18	0.30	0.11	1.742
3	0.06	0.18	0.13	1.279	16	0.24	0.06	0.15	1.636
4	0.06	0.24	0.15	1.388	17	0.24	0.12	0.17	1.662
5	0.06	0.30	0.17	1.236	18	0.24	0.18	0.09	1.877
6	0.12	0.06	0.11	1.582	19	0.24	0.24	0.11	1.817
7	0.12	0.12	0.13	1.613	20	0.24	0.30	0.13	1.511
8	0.12	0.18	0.15	1.595	21	0.30	0.06	0.17	1.319
9	0.12	0.24	0.17	1.591	22	0.30	0.12	0.09	1.602
10	0.12	0.30	0.09	1.799	23	0.30	0.18	0.11	1.645
11	0.18	0.06	0.13	1.651	24	0.30	0.24	0.13	1.619
12	0.18	0.12	0.15	1.747	25	0.30	0.30	0.15	1.231
13	0.18	0.18	0.17	1.596					

**Table 5:** *PEC* Optimization Based on Genetic Algorithm and Numerical Verification.

Parameters	*H*_1_ (mm)	*H*_2_ (mm)	*W*_w_ (mm)	Predicted Value	Numerical Simulation	Relative Error [%]
Value	0.20	0.22	0.09	1.916	1.917	0.05%

**Table 6:** *PEC* Comparison Between Optimization Predictions and Numerical Simulation Results.

Case	*H*_1_ (mm)	*H*_2_ (mm)	*W*_w_ (mm)	$R_{t}$ (K·W^−1^)	$P_{p}$ (W)	ΔP (Pa)	*PEC*
Design in Ref. [19]	0.30	0.30	0.15	0.190	0.031	56,538.498	1.231
Original design	0.20	0.20	0.15	0.176	0.011	20,066.193	1.719
Optimized design	0.20	0.22	0.09	0.161	0.009	17,542.982	1.917

## Data Availability

The original contributions presented in the study are included in the article, further inquiries can be directed to the corresponding author.
